# ‘Doing’ hypertension: Experiential knowledge and practice in the self‐management of ‘high blood’ in the Philippines

**DOI:** 10.1111/1467-9566.13503

**Published:** 2022-08-05

**Authors:** Gideon Lasco, Alicia Renedo, Jhaki Mendoza, Maureen L. Seguin, Benjamin Palafox, Lia M. Palileo‐Villanueva, Dina Balabanova, Martin McKee

**Affiliations:** ^1^ Department of Anthropology University of the Philippines Diliman Quezon City Philippines; ^2^ Development Studies Program Ateneo de Manila University Quezon City Philippines; ^3^ Department of Global Health & Development London School of Hygiene and Tropical Medicine London UK; ^4^ College of Medicine University of the Philippines Manila Manila Philippines; ^5^ Centre for Global Chronic Conditions London School of Hygiene and Tropical Medicine London UK; ^6^ Department of Social and Environmental Health Research London School of Hygiene and Tropical Medicine London UK

**Keywords:** disease ontology, embodiment, hypertension, medical sociology, patient experiential knowledge, symptom

## Abstract

Patients’ embodied experiences do not always correspond to the biomedical concepts of particular diseases. Drawing from year‐long fieldwork in the Philippines that involved semi‐structured interviews, focus group discussions and digital diaries, we examine how individuals ‘do’ hypertension through their embodied experiences and the knowledge and practice that emerge from them. Drawing inspiration from Annemarie Mol’s work on the notion of ‘multiplicity’ of disease, our analysis was informed by a commitment to privileging patients’ embodied experiences and the multiple ontologies of hypertension. We find that for patients diagnosed with hypertension in the Philippines, *symptoms enact illness*; patients rely on their own embodied knowledge to define their illness’ nature (e.g., diagnosis), experience (e.g., frequency of symptoms and non‐chronicity) and praxis (e.g., self‐care practices). We show how this knowledge gained from having embodied experiences of living with the disease interacts in various ways with biomedical knowledge, other diagnostic labels and clinical practices, to shape how hypertension manifests and is managed by patients. Beyond interrogating the relationship between what counts as a ‘disease’ and what is considered a ‘symptom’, our findings underscore the need to pay attention instead to the mutually co‐constitutive processes of embodied experiences and disease categories in co‐producing patient knowledge.

## BACKGROUND

Incorporation of patients’ experiential knowledge is increasingly seen as an essential element of improving the quality of health care (Pomey et al., 2015), training health professionals (Fudge et al., 2008) and shaping health research (Caron‐Flinterman et al., 2005; Carter et al., 2013). Defined as knowledge learnt from living with a disease (Borkman, 1976), including practical knowledge developed to help deal with the disease on a daily basis (Pols, 2010, 2011), this experiential knowledge is increasingly being incorporated into the development of care pathways to ensure that they are patient‐centred (Browne et al., 2010; Wolf et al., 2014). Yet, providers can find the integration of patients’ experiential knowledge with biomedical knowledge and other forms of clinical/medical evidence challenging, partly due to epistemological questions about the legitimacy and authority of embodied experience as a valid form of knowledge (Pols, 2013; Pols & Hoogsteyns, 2016; Renedo et al., 2018). In other words, clinical practitioners can dismiss patient experience and the knowledge that stems from it as ‘subjective’ and therefore not at the same level as ‘objective’ clinical parameters (Renedo et al., 2018; Whelan, 2007). What does taking patients’ experiences seriously mean in practice? In this article we examine embodied experiences of people living with hypertension in the Philippines, focussing on the ontology the symptom as an important source of the patient’s experiential knowledge. We explore how this knowledge that is gained from having embodied experiences of living with the disease interacts in various ways with biomedical/clinical knowledge and practices to shape how hypertension manifests for people living this condition, how people make sense of their illness and how these understandings shape their self‐care practices and ‘therapeutic itineraries’ (Gerhardt, 2006; Mendoza et al., 2021). By ‘embodiment’, we refer to the ‘subjective, private and personal way of knowing the body from the inside’ (Mol & Law, 2004, p. 3) as well as the ways in which this knowledge is enacted alongside biomedical ways of knowing (Gerhardt, 2006; Mol & Law, 2004; Nicholls et al., 2021).

Hypertension remains a major public health concern in the Philippines—one in five adults have elevated blood pressure (Food and Nutrition Research Institute, 2019)—but how people manage it in their everyday lives and what forms of knowledge they use to inform their health‐related behaviour remains under‐explored. Instead, ‘most scholarly attention to hypertension in the Philippines has focussed on biomedical aspects of treatment, epidemiology and clinical outcomes, largely ignoring its social and cultural dimensions’ (Lasco et al., 2020, p. 2).

In this article, we follow and build on the scholarship in medical anthropology and sociology that has explored the ways in which chronic diseases are lived, experienced and enacted (Bury, 1991; Gerhardt, 1990; Manderson & Smith‐Morris, 2010). We draw particular inspiration from the works of Annemarie Mol (2002), who argued that disease is multiple. That is, that instead of just looking at diseases as perceived differently by different people, and problematise distinctions between patients’ and health‐care providers’ views, it is analytically more productive to view diseases as ‘enactments’ of the practices that produce them. Moreover, instead of a priori assuming that there is a disease called hypertension (and in doing so, in the language of Mol, ‘presume that the disease categories of Western medicine are natural’ [Mol, 2002, p. 24]), it is more meaningful to look at the different practices—for example, from measuring blood pressure to attributing bodily sensations as hypertensive symptoms—and how they make‐up—or are thought to make‐up—the condition called hypertension. Applying Mol’s ideas to the case of hypertension, there are multiple clinical ways of enacting hypertension that involve measurements by, for example, oscillometric devices or aneroid sphygmomanometers. Here, hypertension equals particular readings taken by these devices and a particular amplitude of the blood pressure oscillations on the arterial wall; it is not always appreciated that these differ in how they actually measure diastolic and systolic pressure (Babbs, 2012).

While Mol’s focus has been on the enactment of disease by health‐care providers of different specialisations, other scholars have since emphasised how multiplicity extends to different forms of knowledge, particularly those emanating from the patient themselves. As Pols (2013) writes, ‘the knowledge they [the patients] use and develop can be conceptualised as a form of practical knowledge that people use to translate knowledge from different sources (such as medical knowledge) in order to make it useful in their daily life, and how they need to coordinate this knowledge with their other tasks and goals’ (Pols, 2013, p. 83). Our contribution to this perspective is twofold: First, we articulate how this ‘patient knowledge’ emerges from ‘doing’ hypertension, borrowing from Mol’s ideas about ‘doing disease’ (Mol, 2002). That is, hypertension or ‘high blood’ (Lasco et al., 2020) is defined by patients themselves based on their everyday experiences. Second, we problematise the ontological status of ‘symptoms’ in relation to ‘illness’ or ‘disease’: while patients themselves may be ‘medically socialized’ (Pols, 2013, p. 73) to regard their symptoms as a manifestation of, and thus subordinate to, a particular condition, to take their knowledge seriously is to consider their bodily sensations as independent of how these sensations are labelled or framed by health professionals. As we will argue in the discussion, recognising the multiplicity of hypertension in such terms—that is, as lived and practiced by patients themselves in and through their own bodies—is relevant not just for the sociological understanding of chronic illness, but for public health and clinical practice. This is especially true if these disciplines are to adhere to growing calls to put ‘patient perspectives’ (Roberson, 1992), ‘patient knowledge’ (Pols, 2014) or patient ‘experiential knowledge’ at the centre of improvements in care (Blume, 2017).

## METHODS

Part of a larger, mixed‐method longitudinal research that examined pathways and barriers to hypertension care in the Philippines and Malaysia, this study draws primarily on 71 semi‐structured interviews (40 initial, 31 follow up) and secondarily on digital diaries from 40 hypertensive patients aged 35–70 years old residing in low‐income communities in Valenzuela City and Quezon Province in the Philippines, as well as 4 focus group discussions (FGDs) with 33 similar individuals from those same communities. The choice of these two communities was informed by their representativeness of two different contexts—urban and rural—that also correspond to two different health‐care contexts (e.g., different risk factors and levels of access to health facilities). The protocol (Palafox et al., 2018) and the methods used to collect digital diaries (Mendoza et al., 2021) have been published previously. The interviews, diaries and FGDs focussed on the ways in which participants made sense of their illness, while also exploring patient pathways and barriers to care. Interviews typically lasted from 45 to 60 min, while FGDs lasted from 75 to 90 min. Both were conducted in Filipino (Tagalog) by the first and third authors. The follow‐up interviews and the digital diaries collected within a year‐long interval between the initial and follow‐up interviews (in Tagalog) were conducted to explore changes over time in relation to participants’ experiences of living with hypertension, accessing health care and their self‐management practices. Follow‐up interviews allowed us to further explore emergent themes from the initial interviews and issues raised via the digital diaries. While participants’ engagement with the diaries was limited for reasons reported elsewhere—including the lack of seriousness with which the participants generally viewed high blood pressure (Mendoza et al., 2021), the diaries nonetheless provided us with some understanding of how people live with the condition on a daily basis and offered an opportunity to glean some unprompted views about their perceptions of hypertension.

As the first step of an iterative analytical process, inspired by the principles of grounded theory (Charmaz, 2006), open reading and coding of all the data were performed by two of the authors, during which the disjuncture emerged between the plurality of patients’ hypertension‐related practices and bodily manifestations and that of the prevailing biomedical paradigm. Recognising the significance of this observation and its resonances with Mol’s notion of disease multiplicity, we revisited the data paying particular attention to participants’ reported enactments and views of what constituted for them (or did not constitute) their reality of ‘hypertension’. During this second analytical stage, we paid particular attention to the plurality of ways in which hypertension was enacted and ‘materialised’ for participants (i.e., how hypertension manifested through different practices, for example, participants’ actions to respond to symptomatology they associated to hypertension). The major themes that were identified by consensus—*hypertension as diagnosis, hypertension as experience, hypertension as practice*—serve as subheadings of the findings section.

Ethics approval for this study was granted by the Observational Research Ethics Committee at the London School of Hygiene & Tropical Medicine (Ref: 12214) and the University of the Philippines Manila (UPMREB‐2017‐481‐01). All methods were performed in accordance with the relevant guidelines and regulations. All participants gave informed consent.

## FINDINGS

Overall, our participants’ experiential knowledge of ‘hypertension’ or ‘high blood’ is based on their bodily sensations and enacted via self‐care practices such as self‐medication and blood pressure monitoring. Crucially, the biomedical diagnosis of ‘hypertension’ serves as a framework to organise these practices, but ‘hypertension’ is simultaneously (re)made and (re)interpreted through their bodily sensations and care practices.

### (Co‐)constructing hypertension through diagnosis

Clinical diagnosis can construct illness experiences for patients just as much as illness experiences and the sensed body can lead to clinical diagnosis. In other words, for participants the clinical label framed their embodied experiences and led them to adopt particular types of practices to deal with the disease. Bodily experiences also played a key role in prompting engagement with care leading to clinical diagnosis. The same can be said of medical histories, or the ways in which patients narrate their illness. For instance, by asking patients, ‘when did your hypertension begin?’ or ‘when did you know you have hypertension?’, as we did in our interviews, one invites the patient to think in terms of hypertension and interpret their medical biographies accordingly. Consequently, even the very construction of a patient ‘history’ is based on a medical intervention (i.e., the formal diagnosis) serving as a reference point and interpretive frame.

Our research process—which in many ways mirrored a clinical interview in its attempt to establish a ‘history of present illness’—exemplifies this. Our study is explicitly focussed on hypertension, which framed the data collection. As part of the quantitative component of the research, participants had their blood pressure measured. Then, we asked them in our qualitative interviews to recount their experiences with what we *already* called ‘hypertension’ or ‘high blood’. Consequently, if not predictably, participants responded to these questions in those same terms, framing their bodily sensations accordingly, regardless of how such sensations might have been experienced prior to diagnosis. Here, bodily sensations start becoming enactments of the disease for the participant. This conversation with Participant 34, a wife and homemaker from Valenzuela, is illustrative:

Interviewer: Before you were diagnosed with hypertension, have you had any symptoms?

Participant 34: From time to time, when I’m stressed, I would feel some headache and fatigue. But I didn’t take it seriously. But since I was diagnosed, I would take medication whenever I felt those headaches.

Notice the implicit assumption here—made by the interviewer and accepted by the interviewee—that by talking about ‘symptoms’, they were discussing symptoms *of* hypertension. Now part of a shared understanding between the participant and interviewer of the former’s ‘hypertension’, these symptoms are intertwined in the way the patient handles hypertension in their everyday life. The headaches enact the presence of ‘hypertension’ and contribute to the person taking action to deal with the diagnosed disease. The clinical enactment of hypertension—for example, the medication uptake—depends on the reality of hypertension as headache and on how the bodily sensation of the headache is supported now by the diagnostic enactment of the condition. The following passage with Participant 12, a 44‐year‐old resident of Perez, Quezon and recently diagnosed with hypertension, similarly makes such an assumption:

Interviewer: Let’s talk about the time when you were diagnosed with hypertension, what were you feeling then?

Participant 12: My head felt heavy, my neck felt tight, and I was feeling nauseous… I went to the centre and they found out that my blood pressure was high, so I told myself, ‘so I am already “high blood”’.

Interviewer: What about before that day? Do you think that you were already ‘high blood’ without knowing it?

Participant 12: Yes, because I was feeling something in my head and neck.

Interviewer: So you think this has something to do with blood pressure?

Participant 12: I suppose I was really ‘high blood’ because I felt that my face was thick and I had nape pain.

Observe how, for Participant 12, the diagnosis served as a confirmation of what she already knew (e.g., the presence of her symptoms), even as such experiential knowledge (e.g., ‘head felt heavy…neck felt tight’) is only retroactively defined as hypertension because of the diagnosis. Regardless of when a diagnosis of ‘hypertension’ was given, the diagnosis serves as a *post‐facto* confirmation of what previous and concomitant sensations signified. Indeed, regardless of other co‐morbidities, or other factors like stress that might have caused those bodily sensations, they are, because of the diagnosis, interpreted within the framework of clinical hypertension. These bodily sensations (e.g., ‘head felt heavy…neck felt tight’) become sources of knowledge about the presence of the disease and are incorporated as part of the patient’s history. In this way, patients co‐construct their illness through diagnosis (e.g., a high blood pressure reading) and their own symptomatology.

### Sensing hypertension through experience

The above example brings us to a presentation of the ways in which symptoms figure in patients’ experiences and narratives of hypertension. After they are diagnosed with ‘hypertension’, patients continue to interpret their bodily sensations in light of this diagnosis. However, the converse is also true: the diagnosis itself is re‐interpreted in light of specific bodily sensations and becomes dependent on them. Without these embodied experiences, participants do not consider themselves to have ‘hypertension’ or ‘high blood’.

This reliance on symptomatology is illustrated by participants’ narratives of what transpires after diagnosis. Contrary to biomedical understandings of the condition as chronic and largely asymptomatic, participants would describe ‘high blood’ as *pa‐sumpong‐sumpong* (episodic). They would also attribute symptoms, including *masakit ang ulo* (headache), *masama/malabo ang paningin* (blurry vision), *hilo/liyo/lula* (dizziness), *makapal ang batok* (nape tightness), *panghihina* (weakness/fatigue) to this condition. The body acts as a useful ‘*instrument* to diagnose’ the presence of hypertension (Pols, 2011) and to act to self‐manage it (Participant 13 below). The following quotes from two participants are illustrative of how symptoms come to constitute the illness itself:

Participant 06: When I am high blood I get a headache, I feel lula (dizziness) and I feel like vomiting. I can’t fully describe the feeling. But when those things happen to me I know for sure I am high blood.

Participant 13: Sometimes, [high blood] is brought by too much work and heat. By the time I get home at night after I work, because sometimes I work until late, we collect shells in the evening to sell them in the morning, I feel liyo (dizziness) and weak by the time I get home. I take Losartan [medicine to treat high blood pressure] right away then it [high blood] will subside after a while.

Note how for Participant 13, hypertension is brought to life through bodily experiences of dizziness and weakness and how these drive the person promptly to take Losartan, which is then used to lower blood pressure on an as‐needed basis, as opposed to its intended use as a maintenance medication. Indeed, without the presence of these symptoms, Participant 13 would not adhere to a daily regimen of Losartan.

In the absence of such symptoms, many of our participants did not consider themselves to have ‘high blood’ at the time. For instance, Participant 22 did not record diary entries unless he experienced symptoms attributed to hypertension. In one of his digital diary entries, wrote: ‘*Sorry for not texting often. I feel good. My BP is okay at the moment. I’ll text you when I get high blood*’. Clearly, for him, ‘high blood’ is a condition that is transient and its presence is associated with symptoms and sensations that make him feel unwell. Intermittent bodily sensations enact ‘hypertension’ as a non‐chronic disease; as something that comes and goes.

Aside from ‘hypertension’ itself, embodied sensations also figure in the ways in which patients enact related concepts such as cholesterol and stress. This is especially true for ‘high cholesterol’ or oftentimes, just ‘cholesterol’, which participants consider to be closely linked to, if not inseparable from, hypertension (clinicians will understand these as different conditions). The proximity between these two enacted conditions is exemplified by the narratives of our participants, who perceive hypertension as a condition that can be *experienced* or *sensed* through cholesterol in the body, particularly in the blood vessels. The reality of hypertension for the person—for example, its presence or absence; its performance by self‐care practices (e.g., dietary discipline)—depends on fluctuating levels of cholesterol, which can simultaneously depend on particular bodily symptoms (e.g., in the neck as Participant 22 below), as these semi‐structured interview transcripts show:

Participant 19: My main concern is really my cholesterol. I’m afraid it can cause blockage, right? When you have high cholesterol it can lead to stroke… Maybe this is the reason why my BP [blood pressure] is high these days.

Interviewer: Because of the cholesterol?

Participant 19: Yes.

Participant 22: Whenever I eat pork or crab fat, I can feel the cholesterol in my neck. I know that it’s hypertension, but forbidden foods taste best.

Participant 01: I knew that I should not have eaten that food because it was high in cholesterol. True enough, after an hour or so I can already feel ‘high blood’ in my veins… I know it’s my fault when I get high BP. I lose self‐control.

In these accounts, cholesterol itself emerges as a perceptible sensation within the body and contributes to the making and embodiment of ‘hypertension’ for the participants. The sensed ‘cholesterol’ and practices viewed as linked to raised cholesterol (e.g., eating pork) become a way for ‘hypertension’ to manifest. This is further evidenced in this exchange with Participant 42, a 45‐year‐old homemaker from Valenzuela clinically diagnosed with hypertension for 6 years and whose interview narrative about ‘hypertension’ was permeated by references to cholesterol:

Participant 42: [high cholesterol] It’s like feeling a fleshy substance that moves.

Interviewer: I see.

Participant 42: I don’t know if it’s just stress or really cholesterol but it feels like it’s moving inside me. At least that’s how I feel.

Altogether, symptoms and bodily experiences that involve the feeling of ‘high blood’ and ‘cholesterol’ constitute hypertension *as experience.* The co‐dependence of clinical ‘hypertension’ on embodied sensations, and the simultaneous mobilisation of the diagnosis to frame bodily sensations as symptoms or clinical signs of hypertension, structures the temporality of ‘hypertension’ performance for participants, reinforcing—for the most part—a non‐chronic view of the condition. Consequently, bodily sensations also structure self‐care practices, as the next section discusses.

### Enacting hypertension through practice

Individuals’ self‐care practices, for example, including taking anti‐hypertensive medications, having their blood pressure measured or going to a health‐care facility, also stem from their experiential ontology and its reliance on symptomatology. While some patients said they regularly take maintenance medications for hypertension (e.g., amlodipine, losartan), others reported only doing so when experiencing the symptoms outlined above. Otherwise, they may ‘tinker’ with their medications by adjusting the scheduling or dosage depending on bodily experiences of hypertension. Their symptoms become a source for knowledge on how and when to practice hypertension‐related self‐care. For example, Participant 27 (a *sari‐sari* [small variety] store owner from Sampaloc, Quezon) described how she increases her prescribed dosage whenever experiencing symptoms of ‘high blood’:

Participant 27: I have continued taking Amlodipine [An anti‐hypertensive] since 5 years ago.

Interviewer: No change, not even the dosage?

Participant 27: Well, for example, one time, I had high blood, I made it 10 [milligrams], but in normal days I just take 5 [milligrams]. But when I am sinusumpong [experiencing an episode of symptoms], I take 10.

Interviewer: 10?

Participant 27: Yes, 10 mg.

Interviewer: And what do you mean by sinusumpong? What do you feel?

Participant 27: Ayyy, hilo [dizziness]. And pains all over my body.

If experiencing symptoms led some participants to increase their dosage, the converse was also true for others: feeling better can influence their decisions to stop taking medicines. As Participant 8 (a 60‐year‐old variety store owner from Quezon) notes, her ‘forgetfulness’ is based on her self‐assessment of her hypertension going away after resting and seemingly feeling better after that:

Maintenance means I have to take the medicine every day, right? But I forget about it besides my high blood goes away after I take rest.

Clinicians would interpret these practices as illustrative of poor patient expertise, a form of ‘self‐medication’ and non‐adherence, which do not really conform with biomedical prescription of regular intake. For our participants, however, the practices are a logical response to their bodily sensations. These are practices grounded in the embodied knowledge they develop through their experience of ‘hypertension’ and which function as resourceful ways of practicing and dealing with the condition in their everyday life. These embodied practices are also informed by a non‐chronic view of hypertension, which sees the illness as something that is perceptible through their bodies—and one that can be managed accordingly. The following digital diary entries illustrate this point:

Participant 31: I recently stopped my maintenance because I don’t feel high blood these days. It has been a month since I last took Losartan [A commonly prescribed anti‐hypertensive].

Participant 28: My BP is 140/80. I think because I am stressed and I often lack sleep… someone I know is causing me so much stress. I am now taking Stresstabs [A multivitamin brand] to help with stress and sleep.

Aside from medication intake, blood pressure monitoring is likewise performed in these terms, emerging not as a diagnostic process but one that confirms what they already feel, with the measurement devices collaborating in the re‐enactment of ‘hypertension’ for the patient, as the following conversations indicate:

Participant 24: I can feel it when I’m ‘high blood’. Sometimes, I would ask my son to call my friend to check my blood pressure. Last time it was at 160/90. I knew my [blood] pressure is high. After a while my BP was checked again then it became 130. I felt better.

Participant 13: I think I only had high blood four times this December…. I felt very dizzy my head is spinning around [nalula]. So I check my BP to confirm and I am really high blood. I took medicine immediately then after a while I felt okay. I felt that my blood pressure lowered and it did because I checked my blood pressure again.

Participant 20: The doctor tells me that 120/80 is the normal blood pressure. But 130 or 140/90 is my usual BP. I still feel good when I have this blood pressure. I would worry if it goes 150 or higher.

The above passages show that in practice, even clinical indicators are ‘negotiated’ and (re)interpreted by patients to co‐produce their understandings and experience of hypertension, further underscoring the pre‐eminence of embodied experiences in defining illness. Symptoms are not just viewed as manifestations of high blood pressure. They are the very substance of what ‘hypertension’ is. Indeed, what counts as ‘hypertension’ for our participants are those bodily experiences against which measurements are interpreted, negotiated and acted upon (e.g., triggering health‐care seeking behaviours and monitoring of blood pressure).

Finally, consultations are also symptom‐driven; participants mentioned engaging with health‐care services when they felt what they believed to be symptoms of hypertension—typically only when a higher threshold had been breached, for example, a more severe headache. Over the year‐long period during which we engaged with study participants, via repeat interviews and digital diaries, we documented very few instances of patients going for regular follow‐up appointments. Most consultations were either symptom‐driven or incidental (e.g., consulting for another condition). The decision‐making process underpinning such care practices is informed by the participants’ framing of bodily sensations as symptoms of hypertension—as well as their perceptions of what causes them. For instance, as these digital diary entries suggest, some view blood pressure as weather‐dependent and consequently not necessitating actions beyond self‐care, as it will go down as the temperature cools:

Participant 42: I often get my BP checked especially when I feel that my BP is high because I feel nape pain. Maybe it’s because of the extreme heat of the weather right now, and the stress can’t be avoided.

Participant 35: Good afternoon text diary. Thank you very much for reading my entries. Right now it’s very hot. I always have my BP checked right now because I’m scared it might go up suddenly from the intense heat. It can cause severe headache.

These entries show that symptoms like nape pain and headache are linked to explanatory models like heat and stress—and both these symptoms and perceptions determine whether to seek care or to just accept them as part of everyday life (e.g., ‘the stress can’t be avoided’). In this way, symptoms figure not just in determining the existence or presence of illness but in how people cope with it.

## DISCUSSION

### The multiplicity of hypertension

We have paid attention to the ontology of hypertension and the plurality of ways in which this condition is manifested, acted upon and enacted by individuals who have been diagnosed with it. Central to this enactment, as we have illustrated, is the role of embodied experiences, self‐care practices, and at the same time, engagements with the health‐care system through which hypertension *as diagnosis* and *as experience* are co‐produced. Indeed, ‘hypertension’ is brought to life and negotiated through interactions between embodied experiences, the diagnostic label and clinical practices (e.g., blood pressure monitoring), shaping how the condition is experienced and managed by people. We have also illustrated some of the mutually co‐constitutive processes of illness experiences and disease categories. Moreover, we have shown how other diagnostic labels, such as ‘cholesterol’, and linked embodied experiences can contribute to people’s experience and self‐management of hypertension.

The findings make clear that, whether as a diagnostic label, as an embodied experience or as a range of self‐care practices or performances, our participants ‘enact’ hypertension in multiple ways. Beyond local, cultural notions of health that deserve further scholarship especially in non‐western settings (Lasco et al., 2020), there are multiple embodied and practiced ‘presences’ of hypertension (Pols, 2011), which inform our participants’ therapeutic itineraries and their view of their illness as dynamic and non‐chronic.

The findings also resonate with scholarship around the world that finds a strong correlation between patients’ experience of the symptoms they attribute to hypertension and their self‐medication and other self‐care practices (Chen et al., 2009)—even as the timing and duration of these factors (i.e., when exactly do patients self‐medicate or take additional steps to act on their illness) is often overlooked (Rahmawati & Bajorek, 2017). Crucially, far from clashing with clinical paradigms, participants’ enactments of hypertension draw from biomedical constructs in what Mol (2002) calls ‘collaboration’. Indeed, just as people can incorporate blood sugar measurements and bodily sensations in enacting hypoglycaemia (Mol & Law, 2004), our participants likewise incorporate blood pressure measurements, cholesterol levels and their symptoms in enacting, or ‘doing’ hypertension.

The ontological status of ‘symptoms’ as personal resources for patients to realise and deal with disease categories in their everyday life was particularly evident in our participants’ narratives. The corporeal is central to patients’ experiential knowledge of the disease and to their ‘adapted adherence’ (Nicholls et al., 2021) to medication as a ‘situated’ (p. 8) and active form of self‐care. Yet, participants’ narratives also illustrate the role that the hypertension diagnostic label and related clinical practices play in ‘containing’ and ‘identifying’ the disease for patients (Cohn, 2018, p. 249). In biomedical discourse, a symptom has no status on its own—it is always related to illness. There are symptoms of hypertension, symptoms of diabetes, symptoms of flu, but in themselves, these sensations or embodied experiences do not have an independent standing. Any symptom a patient reports to their physician is incorporated by the latter into the former’s medical history and might be attributed to a condition. For instance, nape pain is not regarded as a common symptom of hypertension in the clinical literature and is likely not related to high blood pressure. Nonetheless, the patients in this study experienced hypertension through, and in relation to, bodily sensations such as nape pain. A more stark example, also presented above, is the sensation of ‘cholesterol’ in the blood, which—given its absence in biomedical formulations of hypercholesterolaemia or hypertension—further challenges what ‘symptom’ means. Can the diagnostic label of ‘hypercholesterolaemia’ be dissociated from the bodily sensation that the patients associate with it? Regardless, these concepts figure in our participants’ bodily enactments of what counts for them as ‘high blood’ or ‘hypertension’.

Indeed, our study shows that far from serving as indicators of a certain disease, *symptoms enact the illness*, and this may be particularly important for asymptomatic chronic diseases, as well as for those with multiple conditions but whose symptoms may lead patients and providers alike to conflate these symptoms into one entity. But at the same time, the *illness also enacts the symptoms*, as we have seen in the ways patients came to think of the experience of ‘cholesterol’, headache, nape pain or dizziness in terms of ‘high blood’. Again, this is illustrative of the ‘collaboration’ that inevitably ensues in chronic illness.

The multiplicity of hypertension is not just a theoretical argument; it has clinical implications. The insistence in making people’s experience fit within just one ontological mode of ‘hypertension’, for instance, can marginalise or subordinate people’s embodied experiences in favour of symptoms and narratives that more neatly fit how hypertension is ‘made’ in textbooks or clinics. Instead of treating symptoms merely as diagnostic clues that make‐up a disease, they must be taken seriously, not least by actually trying to address them (e.g., through medications, non‐pharmacologic interventions) regardless of what they might indicate.

Moreover, such an insistence can make clinicians and public health practitioners lose sight of the ‘tinkering’ that is done by patients on every aspect of their ‘care’ (Mol et al., 2015)—from the dosage of medicines to the interpretation of blood pressure measurements—that is driven by their experiential knowledge, including one that is developed and shared with peers (Kingod, 2020; Pols & Hoogsteyns, 2016). If the goal is hypertension control, then it is easy to see how such self‐care practices can undermine its achievement, unless providers work to bridge the gap between their own professional knowledge and their patients’ experiential embodied knowledge, identifying these practices and understanding what they mean for their patients, instead of assuming that ‘normal blood pressure’ means the same thing for everyone.

Such fuller engagement with patients’ embodied experiences (Williams, 2006) also presents a more meaningful way of operationalising a more patient‐centred model of care. This ‘dialogue’ between the sociology and biology/biomedicine of hypertension requires recognition of patient voices as legitimate in the health‐care relationship and as useful sources of evidence to shape care (Williams, 2006). This kind of engagement also needs to recognise embodied positions and the role of patient’s bodies as sources of practical knowledge to live with a disease (Pols, 2010, 2011). While this is applicable everywhere, it is arguably even more relevant in settings like Southeast Asia where the doctor–patient relationship has been characterised as ‘one‐way’ and ‘paternalistic’ (Claramita et al., 2013). Such an approach entails moving away from thinking of hypertension as a ‘separate entity’ (Cohn, 2018, p. 254) and instead calls for considering how the increasing occurrence of multi‐morbidities might broaden the ‘constellations’ of diagnostic categories (Cohn, 2018). Can patients’ experiential ontologies be mobilised as therapeutic Allies, for instance in framing maintenance medications as form of symptom prevention—and not as a means of control for something that for them does not exist? And can such privileging of embodied experience lead to a more holistic patient‐centred approach to care? Just as our article suggests that automatically linking symptoms to particular diseases can lead to fragmentation of care, taking them seriously, independent of what they might clinically signify, can help avoid obscuring the complexities of illness experiences and bring about improvements in care.

To be sensitive to patients’ own enactments of whatever it is that is bothering them, we join Mol in recommending ethnographic methods, given their ability to ‘foreground practices and draw together disparate entities in a single story’ and ‘produce rich stories of lived bodies in which medicine figures as a part of daily life’ (Mol, 2002, p. 58). Just as untethering symptoms from illness is key to seeing patients’ enactments of hypertension, untethering illness from their lives can yield multiple ontologies of care (Ivanova et al., 2016), and hopefully, a multiplicity of therapeutic possibilities; if nothing else, a more ontologically sensitive, ethnographically informed dialog with their patients and their experiential knowledge (Renedo et al., 2018) and forms of self‐care (Pickard & Rogers, 2012).

Our research had some limitations. For instance, our study design did not allow for further exploration of other forms in which the multiplicity of hypertension might take place, for instance, in the realm of medications (Danholt & Langstrup, 2012). We drew on reported data from interviews, focus groups and ‘digital diaries’ and would have benefited from conducting ethnographic observations to further explore how individuals and families ‘do’ the ‘homework’ (Mattingly et al., 2011) that chronic illnesses require—especially since, as we have noted in the findings, the research process can contribute in co‐constructing enactments of hypertension by framing the patient’s experiences in relation to it. It would also be useful to observe different kinds of health‐care provision and care encounters and how they ‘enact’ hypertension—from general practitioners to specialists—as well as to look for other demographics (e.g., younger populations, higher‐income households).

## CONCLUSION

These limitations notwithstanding, we have gleaned some important insights that have theoretical and clinical significance. While the significance of patient experiences has long been recognised in the literature, we have shown the central role that symptoms play in giving meaning and form to these experiences. Indeed, *symptoms enact illness f*or patients diagnosed with hypertension in the Philippines, even as, by providing (or imposing) conceptual categories, the *illness also enacts symptoms*. We have also shown that this experiential knowledge is ‘hybrid’ instead of being opposed to or distinct from biomedical knowledge: Symptoms and diagnostic labels co‐produce and co‐constitute each other.

To go back to the question raised in the introduction, taking patient experience seriously means that we must untether symptoms from the conditions they are associated with, and see them not just as diagnostic clues but therapeutic targets. Indeed, avoiding glossing over patients’ embodied positions in care encounters is a more useful and potentially empowering approach for patients to become actively involved in the day‐to‐day self‐management of their condition (Pols, 2011). If health‐care professionals were to withhold imposing conceptual categories on patients and interlocutors, then perhaps they could better ‘collaborate’ not just in terms of what for them constitutes ‘illness’, but also what constitutes ‘care’.

## AUTHOR CONTRIBUTIONS

**Gideon Lasco**: conceptualisation (equal); methodology (equal); writing—original draft (lead); formal analysis (lead); writing—review and editing (equal). **Alicia Renedo**: conceptualisation (equal); formal analysis (supporting); writing—review and editing (equal). **Jhaki Mendoza**: writing—original draft (supporting); methodology (equal); formal analysis (supporting); writing—review and editing (supporting). **Maureen Seguin**: formal analysis (supporting); writing—review and editing (supporting). **Benjamin Palafox**: formal analysis (supporting); writing—review and editing (supporting). **Lia Palileo‐Villanueva**: supervision (equal); formal analysis (supporting); writing—review and editing (supporting). **Dina Balabanova**: supervision (supporting); formal analysis (supporting); writing—review and editing (supporting). **Martin McKee**: supervision (equal); formal analysis (supporting); writing—review and editing (equal).

## Data Availability

The interview transcripts are available on request, and subject to gaining ethics approval, from the authors.
